# Antimicrobial Stewardship for Africa

**DOI:** 10.1093/jacamr/dlz006

**Published:** 2019-04-08

## Abstract

**Graphical Abstract:**
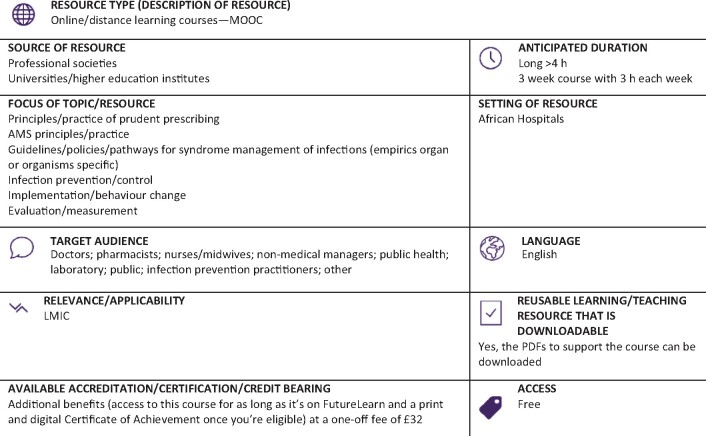

**Resource web link: https://www.futurelearn.com/courses/antimicrobial-stewardship-for-africa** (Full classification scheme available at: http://bsac.org.uk/wp-content/uploads/2019/03/Educational-resource-review-classification-scheme.pdf)

**WHO region and country (World Bank):** AFRO, Nigeria (LI, LMIC)

## Peer review commentary

Overall the course is excellent, the material is well presented and well tailored for LMICs. The course outline and progression are appropriate and the use of local African antimicrobial resistance (AMR) experts that participants can identify within their local settings enriches this antimicrobial stewardship (AMS) programme. The content presents the realities of clinical practice in LMICs and the experts did very well in defining AMS and answered the why, what and how of implementing AMS in different settings.

The introduction sets the scenario for the importance of AMS in an African hospital context very well. The drivers are highlighted and the course participants are challenged on what they can do to address AMS. The simplicity of presentation created by Africans makes the subject matter that participants can relate to. The additional YouTube video helps to amplify the problem.

Using a story healthcare workers (HCWs) can relate to has been quite effective in presenting aspects of AMS and answering the questions why HCWs should implement AMS in health facilities. It is a story most of us, if not all HCWs, can relate to. Indiscriminate, including suboptimal, use of antibiotics leads to stock outs, high prices, longer hospital stays, comorbidities and death. Lack of knowledge and guidelines was also well illustrated. The strengths of the course are as follows:

- The course has a very good outline, broken down into 3 weeks with topics that build on each other, is well-paced and can be taken at any time. The introduction sets the background and scene to AMS very well.
- The course is evidence- and science-based and is in line with recommendations of professional bodies, societies and intergovernmental organizations such as WHO.
- The case studies used are appropriate to the setting and context augmented by the use of a combination of drama (with the transcript available) and self-reading.
- The videos carry very clear messages on each sub-thematic subject and are short (5–10 min) allowing for ease of watching and reading.
- Further reading materials that can be accessed by the links provided are valuable although not all references appear to have links, for example the Data Susceptibility Testing and microbiology identification in the African context refers to CDDEP for further reading but the link is missing.
- The comments section following each video provides a very good means of engaging with other course participants, something that is evident.
- The presentations are excellent in terms of scientific accuracy and understanding the challenges faced in African countries and provide practical ways of addressing AMS in low-resource settings.
- Examples from Nigeria and South Africa present the participants with many practical lessons that they can learn from and apply in their healthcare facility settings.
- The weekly summaries help to emphasize the major concepts and points discussed in the previous week and present a good way of ending the week.
- The presentation by Prof. Mehtar is another excellent presentation in this course as it addresses most questions we encounter in African countries: how and where do we start to implement a stewardship programme.
- One of the highlights of this course is showing the important linkages between AMS and infection prevention and control (IPC) thereby facilitating the local need for IPC and AMS teams and resources to work together towards common goals.
- Rating of the week’s course is helpful for reviewing but also allows participants to reflect and might trigger them to go back and review where they may have missed the material.
- The audit and feedback session may be challenging for some especially where the calculations are concerned.
- The article and presentation on feedback and self-monitoring as effective behavioural change techniques were well presented and articulated. This is an area that often is not presented nor done in health facilities to support stewardship efforts. Closing the loop of audits to feedback is essential towards behavioural change.
- The end-of-course summary is excellent and the provision of more resources such as the e-book on Antimicrobial Stewardship: From Principles to Practice.

A number of challenges were identified as well as opportunities for development:

- The course appears to lean heavily towards doctors, nurses, nurse practitioners and pharmacists and might miss out the other HCWs [such as ancillary healthcare practitioners (HCPs)] because of the language and the terminology that is used. For example, in the clinical case study has a few medical terms that ancillary HCPs may not be familiar with e.g. anastomotic leak, laparotomy.
- The mechanics of moving or going back to some previous sections is not very user friendly. For example, moving back to Unit 1 from Unit 3 or a referenced section you might have a link to like 2.3, you lose the current section you are working on.
- The MOOC requires internet connection which will be a big challenge in institutions outside of the main cities to complete the course, limiting its reach. Where intermittent internet is present participants will face challenges connecting. This is something that online courses will need to address for improving longer-term sustainability and reach of such courses.
- Although most presentations are very clear, some PowerPoint presentations with charts and diagrams are not clear. For example, Alaal Afda’s presentation is excellent but some PowerPoint slides are not clear. The authors of the programme should address these.
- Institutions and participants should be encouraged to have staff take the course in groups to maximize team learning and the ‘let’s talk’ segments which would allow for group discussions.

